# Characterization of *Bombyx mori* and *Antheraea pernyi* silk fibroins and their blends as potential biomaterials

**DOI:** 10.1007/s40204-016-0057-3

**Published:** 2016-10-27

**Authors:** Shuko Suzuki, Traian V. Chirila, Grant A. Edwards

**Affiliations:** 1 Queensland Eye Institute, South Brisbane, QLD 4101 Australia; 2 Science and Engineering Faculty, Queensland University of Technology (QUT), Brisbane, QLD 4001 Australia; 3 Faculty of Medicine and Biomedical Sciences, The University of Queensland (UQ), Herston, QLD 4029 Australia; 4 Australian Institute of Bioengineering and Nanotechnology (AIBN), The University of Queensland (UQ), St Lucia, QLD 4072 Australia; 5 Faculty of Science, The University of Western Australia (UWA), Crawley, WA 6009 Australia

**Keywords:** *Bombyx mori* silk, *Antheraea pernyi* silk, Silk fibroins, Membranes, Mechanical properties, FTIR analysis

## Abstract

Fibroin proteins isolated from the cocoons of certain silk-producing insects have been widely investigated as biomaterials for tissue engineering applications. In this study, fibroins were isolated from cocoons of domesticated *Bombyx mori* (BM) and wild *Antheraea pernyi* (AP) silkworms following a degumming process. The object of this study was to obtain an assessment on certain properties of these fibroins in order that a concept might be had regarding the feasibility of using their blends as biomaterials. Membranes, 10–20 μm thick, which are water-insoluble, flexible and transparent, were prepared from pure fibroins and from their blends, and subjected to water vapor annealing in vacuum, with the aim of providing materials sufficiently strong for manipulation. The resulting materials were characterized by electrophoretic analysis and infrared spectrometry. The tensile properties of the membranes were measured and correlated with the results of infrared analysis. At low concentrations of any of the two fibroins, the mechanical characteristics of the membranes appeared to be adequate for surgical manipulation, as the modulus and strength surpassed those of BM silk fibroin alone. It was noticed that high concentrations of AP silk fibroin led to a significant reduction in the elasticity of membranes.

## Introduction

Silks are biopolymers with a wide range of mechanical properties, which are produced by certain organisms such as insects and spiders. The silk cocoons generated in the pupation stage by the larvae of domesticated silk moth *Bombyx mori* (family *Bombycidae*) have been a source for textile fibers for millennia. Closer to our times, the two main constitutive proteins (fibroin and sericin) of the silk produced by *B. mori* silkworm and a few other silkworms of the class Insecta have been investigated also as potential biomaterials (Altman et al. 2003; Vepari and Kaplan 2007; Hakimi et al. 2007; Kundu et al. 2008; Wang et al. 2009; Murphy and Kaplan 2009; Harkin et al. 2011; Pritchard and Kaplan 2011; Sehnal 2011; Wenk et al. 2011; Chirila et al. 2013; Kundu et al. 2013; Hodgkinson and Bayat 2014; Khan and Tsukada 2014; Kundu et al. 2014; Patra and Engel 2014; Wang et al. 2014; Koh et al. 2015; Lamboni et al. 2015; Cao and Zhang 2016; Kapoor and Kundu 2016). This type of application implies their direct contact with the human living tissue; consequently, the ability of fibroin or sericin to function as a nontoxic substratum for the attachment and growth of cells specific to the host tissue is essential (Minoura et al. 1990, 1995a, b). *B. mori* silk fibroin (BMSF) has been by far the most investigated silk substratum, on which a variety of cells have been cultured in vitro successfully (Minoura et al. 1990, 1995a, b; Wang et al. 2006; Chirila et al. 2008).

In spite of generally satisfactory growth of cells reported on BMSF, the adhesion of cells to its surface appears to be rather a non-specific process, as this protein does not include any of the known recognition ligand peptide sequences for integrins (the main receptors mediating cells’ anchorage to substrata). A potential strategy to enhance the cell adhesion to BMSF is to mix it with the fibroin isolated from a wild silkworm, *Antheraea pernyi* (family Saturniidae), henceforth APSF. The latter was shown to contain the adhesion peptide sequence arginine-glycine-aspartic acid (RGD), which is a typical ligand peptide motif for the integrin receptors on the cell surface. An earlier report showed that APSF promoted indeed a better attachment of cells than BMSF (Minoura et al. 1995a). More recent studies (Bray et al. 2013; Hogerheyde et al. 2014), involving human corneal cells, indicated acceptable cell attachment to APSF or to BMSF/APSF blends, however, an enhancement induced by APSF could not be unequivocally proved.

In this study, the pure fibroins were isolated by first removing the sericins from the silk threads, and then a range of BMSF/APSF blends were prepared, respectively, from these sericin-deprived, “regenerated” silks, and cast as membranes. The enhancement of cell adhesion to the fibroin membranes is important for tissue engineering applications, but not sufficient. Equally important is the strength of these membranes, as they will ultimately be manipulated by surgeons to be implanted. We evaluate and discuss here the characteristics of membranes made of BMSF, APSF and their blends, with an emphasis on their mechanical properties.

## Materials and methods

### Materials

The *B. mori* silkworm cocoons were provided by Tajima Shoji Co. Ltd. (Yokohama, Japan), and the *A. pernyi* cocoons by the Lepidoptera Breeders Association (Sleaford, UK). All chemicals were purchased from Sigma-Aldrich (St Louis, MO, USA). Water of high purity (Milli-Q or equivalent quality) was used in all experiments. Minisart^®^ filters (0.2 μm) and Minisart^®^-GF pre-filters (0.7 μm) were supplied by Sartorius Stedim Biotech (Göttingen, Germany). The dialysis cassettes (Slide-A-Lyzer^®^) (MMCO 3.5 kDa) were supplied by Thermo Scientific (Rockford, IL, USA), and the dialysis tubing with MMCO 12.4 kDa by Sigma-Aldrich.

### Preparation of regenerated fibroin solutions

The BMSF solution was prepared according to a protocol previously reported (Chirila et al. 2008), which led to a concentration of 1.78% w/v BMSF (by gravimetric analysis). To obtain the APSF solution, the cocoons were dried, cut into approximately 1 cm **×** 1 cm pieces, weighed and then placed in 1 L boiling solution of sodium carbonate containing 0.85 g of salt for 1 g of cocoon material. After 1 h of boiling, the fibrous material was squeezed to remove the excess liquid, and then treated for 20 min, three times in succession, in 1 L of warm (60–70 °C) water, followed by drying in a fume hood for at least 12 h. The dry fibrous mass was mixed with neat calcium nitrate tetrahydrate (20 times excess to the amount of fibers) at 105 °C and kept for 5 h on an oil bath while stirring very slowly. The resulting solution was injected into pre-treated dialysis tubing (MMCO 12.4 kDa), which was placed into a 1-L beaker with chilled water (4 °C) and kept in a refrigerator. Water was exchanged for fresh pre-chilled water 6 times at increasing intervals over 3 days of dialysis. The resulting fibroin solution was removed carefully from the dialysis tubing and filtered successively through 0.7- and 0.2-µm filters into a dialysis cassette (MMCO 3.5 kDa) in pre-chilled 30% w/v solution of poly(ethylene glycol) (MM 10 kDa), and left to dialyze for ~10 h. The solution collected from the dialysis cassette contained 1.50% w/v APSF as determined by gravimetric analysis.

### Preparation of fibroin membranes

The BMSF and APSF membranes were cast from the solutions produced as described above. To make the blends, the two fibroin solutions were mixed together to provide mixtures with the following compositions (BMSF/APSF, in % w/w): 90/10, 70/30, 50/50, 30/70 and 10/90. Prior to casting, all solutions were allowed to homogenize in a refrigerator for 3 h. The solutions were then poured into 45-mm glass Petri dishes, which were placed in a fan-driven oven and kept for 12 h at 25 °C. After drying, the membranes were placed in a vacuum enclosure, where they were annealed at –80 kPa in the presence of water (in a beaker) without heating. The annealing duration for the membranes rich in BMSF (70, 90 and 100%) was 6 h, while for the others was 24 h. The annealing process induced complete insolubility in water.

The thickness of the dry membranes, measured with an upright stand type micrometer (US-16B, TECLOCK, Japan), was 15 ± 5 μm. The membranes were transparent, insoluble in water, and displayed the characteristics of a flexible hydrogel.

### Gel electrophoresis analysis

The molecular mass distribution in each of the two fibroins was investigated by sodium dodecyl sulphate–polyacrylamide gel electrophoresis (SDS-PAGE). A Novex^®^ XCell Sure Lock™ Mini-Cell system (Life Technologies Inc, Carlsbad, CA, USA) and an EPS-250 Series II Power Supply unit (CBS Scientific Company Inc, San Diego, CA, USA) were employed. The fibroin membranes (prepared as described in the previous section) were solubilized either in a solution of lithium bromide (the BMSF membranes) at 60 °C, or in neat calcium nitrate at 105 °C (the APSF and BMSF/APSF blended membranes), and the resulting solutions were dialyzed and filtered. Each solution was mixed with both NuPAGE^®^ sample preparation reagent and NuPAGE^®^ sample reducing agent, and heated at 70 °C for 10 min. A volume of each solution containing about 50 μg protein was loaded into a 1-mm thick 3–8% NuPAGE^®^ Novex^®^ Tris–Acetate gel in NuPAGE^®^ Tris–Acetate SDS Running Buffer. The gels were run at a voltage of 150 V for 1 h together with a HiMark™ Pre-stained Protein Standard (Life Technologies). The gel was then washed in three 5-min stages with distilled water, and soaked in SimplyBlue™ SafeStain solution containing Coomassie^®^ G-250 stain for 1 h under gentle stirring. The resulting gel was washed in distilled water for 1 h and then photographed.

### Mechanical testing of the membranes

Strips (1 cm × 3 cm) were cut out from each membrane and subjected to tensile measurements (for stress, modulus and elongation) in an Instron 5848 microtester (Instron, UK), equipped with a 5 N load cell. The gage distance was set to 14 mm. The samples were loaded by pneumatic grips and submersed in phosphate buffer solution (pre-heated to 37 ± 3 °C) in a BioPuls™ unit for 5 min prior to stretching. Stress–strain plots were generated during the measurements, and the Young’s moduli were computed in the linear region. Elongation at break was also measured. The mean values were calculated from results generated by six measurements for each membrane, which were cut out from at least three different membranes for the same composition. The results were processed by the one-way analysis of variance (ANOVA) in conjunction with Tukey–Kramer multiple comparisons test using the GraphPad InStat^®^ Version 3.10.

### Analysis by Fourier transform infrared-attenuated total reflectance (FTIR-ATR) spectrometry

The FTIR-ATR spectra were recorded in a Nicolet Nexus^®^ 5700 FTIR spectrometer (Thermo Electron Corp., Marietta, OH, USA) equipped with a diamond ATR accessory and the OMNIC 7 software package. Each spectrum resulted by the co-addition of 64 scans in the range 4000–525 cm^−1^ at a resolution of 8 cm^−1^.

## Results and discussion

While the literature dedicated to mechanical properties of BMSF is extensive, the APSF has been considerably less investigated. A survey of the relevant literature on APSF showed that structure, thermal properties, spectrometric characteristics and crystallization patterns were the topics of main interest, rather than its mechanical properties. The latter have been reported in only one article (Fu et al. 2011), and restricted to the native AP fibers extracted from mature larvae’s glands. Although the regenerated APSF has been also prepared and studied (Kweon et al. 2000; Zuo et al. 2009), its mechanical properties were not assessed. Structurally, APSF appeared to be closer to the fibroin produced by spiders (spidroin) than to BMSF. In fact, studies on BMSF/APSF films cast either from fibroins collected from the larvae’s glands (Tsukada et al. 1994) or from regenerated fibroins (Wu et al. 2012) have suggested some physical incompatibility between these two proteins due to crystalline phase separation, but no mechanical evaluation was carried out to corroborate this observation.

**Fig. 1:**
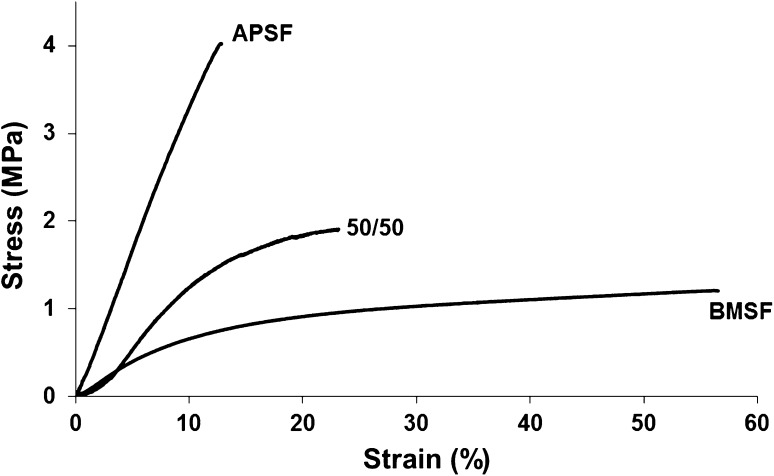
Stress-strain plots for BMSF, APSF and their equivalent blend (50:50 by weight)

We are developing in our laboratories silk fibroin membranes as substrata for growing ocular cells (corneal or retinal) to create fibroin-cells constructs to be used in the treatment of blinding conditions caused by trauma or disease (Chirila et al. 2008, 2016; Harkin et al. 2011; Madden et al. 2011; Shadforth et al. 2016). The successful use of implants made of silk fibroins is critically related to the tensile stress applied during surgical manipulation. There is a large body of evidence, including our experimental results, that BMSF as such has mechanical properties superior to almost all other biopolymers proposed or used as biomaterials. Considering the absence of reports on mechanical characteristics of membranes made from BMSF/APSF blends, we have carried out such measurements in this study. An illustrative stress–strain plot is shown in Fig. 1, and the results of complete mechanical evaluation for all samples are given in Table 1.

**Table 1:** Tensile characteristics of membranes made of BMSF, APSF and their blends (mean ± SD, *n* = 6)

Composition BMSF/APSF (% w/w)	Young’s modulus (MPa)	Ultimate strength (MPa)	Elongation at break (%)
100/0	12.9 ± 3.5^a^	1.7 ± 0.6	58.0 ± 22.3
90/10	17.2 ± 2.0^a^	2.0 ± 0.4	51.7 ± 16.7
70/30	17.7 ± 2.3^a^	2.1 ± 0.3	43.7 ± 14.4
50/50	15.4 ± 6.5^a^	1.4 ± 0.4^b^	28.8 ± 9.4
30/70	22.8 ± 6.9	2.3 ± 1.2	19.7 ± 9.7
10/90	32.2 ± 6.1	2.7 ± 0.7	15.3 ± 2.3
0/100	36.4 ± 8.8	3.2 ± 1.9	14.3 ± 7.4

^a^These values are statistically different (*p* < 0.001) from those measured for the membranes containing 90 and 100% w/w APSF

^b^This value is statistically different (*p* < 0.05) from that measured for 100% w/w APSF

Comparing BMSF and APSF as such, the latter membranes were stiffer and stronger. In their blends, we noticed a decrease in both modulus and ultimate strength in the region of equal concentrations of the two components, while they increase in the regions of either low BMSF or low APSF. This is consistent with the observation (Tsukada et al. 1994) that in BMSF/APSF blends the crystalline compatibility is high at low concentrations (about 20% w/w or less) of either component, but crystalline phase separation occurs when their concentrations are similar (e.g. in the range 40–60% w/w of any of these components). It is believed (Hu et al. 2011) that an enhanced crystallinity in silk fibroins leads to enhanced rigidity and strength, therefore, a reduced crystallinity due to phase separation could cause lower modulus and tensile strength. Our results appear to confirm the above assumptions.

There was a dramatic reduction of the elongation at break (Table 1) as the proportion of APSF in blends increased, an obvious consequence of the enhanced stiffness of this component (its modulus is about 3 times higher than that of BMSF).

**Fig. 2:**
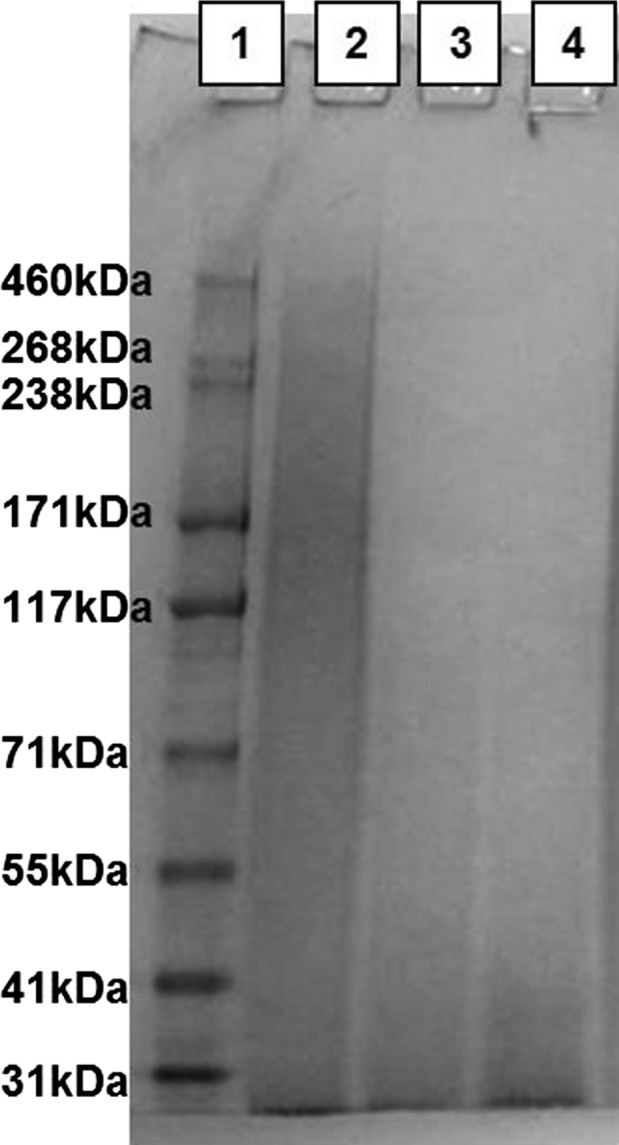
Electrophoretic patterns of BMSF, APSF, and their equivalent blend: *1* molar mass marker positions; *2* BMSF; *3* blend BMSF/APSF 50/50% w/w; *4* APSF. (See text for details of analysis)

Electrophoretic mobility of the polypeptide components in each fibroin was investigated by SDS-PAGE with an aim to reveal the distribution of their molecular masses. The analysis showed (Fig. 2) typical smear patterns for BMSF, APSF and their blend, such indicating that the original polypeptides were degraded during degumming due to hydrolytic reactions at the elevated temperatures and pH values employed in the process. It is known that in these conditions BMSF can be extensively degraded (Trefilleti et al. 1980; Zuo et al. 2006), and this can be assumed for APSF too. Our analysis clearly showed (Fig. 2) that the hydrothermal degradation of APSF was more advanced than that of BMSF, likely due to higher temperatures involved in the processing of the former. This, in turn, may contribute to the significant decrease in elongation and increase in stiffness of the blends with high content of APSF.

**Fig. 3:**
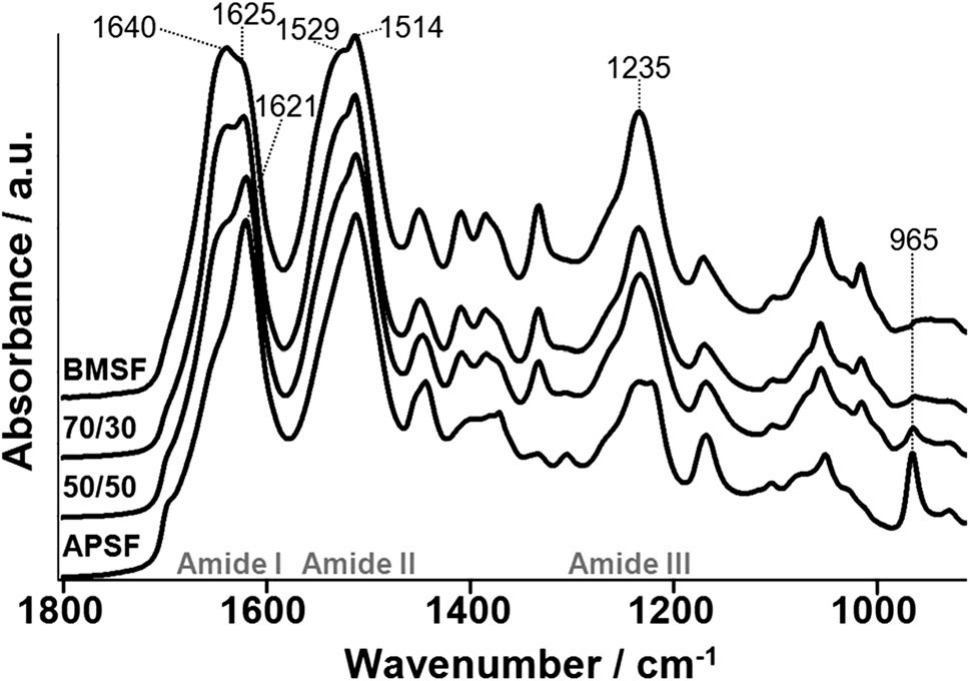
The FTIR-ATR spectra of BMSF, APSF and two of their blends (70/30 and 50/50% w/w)

The membranes were also analyzed by FTIR-ATR spectrometry with an aim to gain some insight regarding the relation between mechanical properties and the secondary structure of the silk proteins. Figure 3 shows the spectra of BMSF, APSF and their blends in the respective proportions of 70/30 and 50/50% w/w. BMSF displayed strong absorption bands in the Amide I (1640 cm^−1^), Amide II (1529 cm^−1^), and Amide III (1235 cm^−1^) regions, which are commonly attributed to random coil conformation in the amorphous regions of the protein, and also at 1625 and 1514 cm^−1^ corresponding to β-sheet structures in the crystalline region (Magoshi and Magoshi 1977; Tsukada et al. 1994; Lu et al. 2011). Comparatively, in the spectrum of APSF, the strong absorption bands at 1621, 1514 and 1235 cm^−1^ indicated a higher β-sheet content, which is supported by an absorption at 965 cm^−1^ attributable also to β-sheet structures (Kweon et al. 2000; Li et al. 2003). The spectra of blends showed bands characteristic to the two fibroins, with the band intensities corresponding to the BMSF/APSF ratios, and no additional absorption bands. This finding is in agreement with a previous study (Tsukada et al. 1994), where it was interpreted as a result of very weak, or even absent, molecular interactions between the two fibroins. This may be, indeed, related to the quantitative decline in the mechanical properties when none of the two fibroins is predominant in the blend.

## Conclusions

The membranes made of blends of BMSF and APSF display adequate mechanical strength and stiffness when any of the two components is quantitatively predominant in the formulation. However, when the proportions of BMSF and APSF are similar, the strength and modulus are lower due to possible crystalline phase separation in this region. This aspect, and also the decrease in their elasticity induced by increasing the amount of APSF, shall be considered when selecting the most appropriate formulation for a membrane that will have to be surgically manipulated during tissue engineering applications.

### **Authors’ contributions**

TVC: designed and coordinated the study; wrote the manuscript; contributed to the interpretation of results. SS: carried out the production and analysis of silk fibroins; drafted sections of the manuscript; executed and organized the graphic material in the manuscript; reviewed the final draft. GAE: contributed to mechanical analysis of fibroins and to interpretation of results; reviewed the final draft.

## Abbreviations

BMSF: *Bombyx mori* silk fibroin
APSF: *Antheraea pernyi* silk fibroin
FTIR-ATR: Fourier transform infrared-attenuated total reflectance
MM: Molecular mass
MMCO: Molecular-mass cut-off
SD: Standard deviation
SDS-PAGE: Sodium dodecyl sulphate–polyacrylamide gel electrophoresis
